# Diagnosis Accuracy and Prognostic Significance of the Dickkopf-1 Protein in Gastrointestinal Carcinomas: Systematic Review and Network Meta-analysis

**DOI:** 10.7150/jca.49970

**Published:** 2020-10-18

**Authors:** Xiaowen Jiang, Fuhai Hui, Xiaochun Qin, Yuting Wu, Haihan Liu, Jing Gao, Xiang Li, Yali Xu, Yingshi Zhang

**Affiliations:** 1 Department of Life Science and Biochemistry, Shenyang Pharmaceutical University, Shenyang, 110016, China.; 2 Department of Pharmacy, Shenyang Pharmaceutical University, Shenyang, 110016, China.

**Keywords:** DKK-1, Gastrointestinal carcinomas, Diagnosis, Prognostic, Network meta-analysis

## Abstract

**Objective:** To evaluate the diagnosis accuracy and prognostic significance of bio-marker dickkopf-1(DKK-1) protein in GIC, and also sub-type of hepatocellular carcinoma (HCC), pancreas carcinomas (PC), oesophageal carcinoma (EPC) and Adenocarcinoma of esophago-gastric junction (AEGJ), etc.

**Methods:** Electronic databases were searched from inception to May 2020. Patients were diagnosed with gastrointestinal carcinomas, and provided data on the correlation between high and low DKK-1 expression and diagnosis or prognosis.

**Results:** Forty-three publications involving 9318 participants were included in the network meta-analysis, with 31 of them providing data for diagnosis value and 18 records were eligible for providing prognosis value of DKK-1. DKK-1 has a moderate diagnostic value for overall GIC, HCC and PC. In addition, for the combined diagnosis value of DKK-1 +AFP, high diagnostic accuracy value could be determined in HCC and early HCC group, respectively. Whereas, diagnosis efficiency of DKK-1+CA19-9 was also better than that of DKK-1 alone with AUC value is above 0.95. For the prognosis meta-analysis of histopathological stratification, we found that EPC and AEGJ ranked the best for the histopathological stratification of prognosis from network meta-analysis. This systematic review protocol was registered with the PROSPERO registry (No.CRD42020167910).

**Conclusion:** DKK-1 has good diagnostic accuracy, especially combination of DKK-1+AFP in HCC and DKK-1+CA19-9 in PC, whereas modest prognostic significant in GIC. Future head-to-head researches are warranted for DKK-1 expression in HCC and PC tissue.

## Introduction

Gastrointestinal carcinomas (GIC), mainly including liver carcinoma (LC), pancreatic carcinoma (PC), gastric carcinomas (GC), oesophageal carcinoma (EPC) and colorectal carcinoma (CRC), are the causes of high morbidity and mortality worldwide^1^. In addition, GIC represents a significant health burden in society, and its prevalence is inclined to continue to grow in the future^2^. LC contains two types of histopathology, hepatocellular carcinoma (HCC) and intrahepatic cholangiocarcinoma (IHCC), with a proportion of HCC is greater than 95%^3^. GIC has the characteristics of a high degree of differentiation, concealed early onset, rapid development in the middle stage, and easy metastasis and recurrence in the late stage; thus, most patients are in the middle and late stages when clinically diagnosed^4^. Therefore, it is extremely important to find a biomarker with better diagnostic value to diagnose GIC early, as it is very important to improve the overall survival of GIC patients. It is expected to have a better predictive effect of histopathological stratification to predict the evolution and prognosis of patients' condition, which is of great significance for the overall control of patients' evolution and prognosis changes^5^-^6^.

Dickkopf-1 (DKK-1), a secreted glycoprotein that acts as an antagonist of the Wnt/β-catenin signalling pathway, is part of the DKK family of proteins that includes Dkk-2, Dkk-3 and Dkk-4. The Wnt/β-catenin signalling cascade governs cell proliferation and cell fate during embryonic development and tissue homeostasis^7^-^8^. The Wnt/β-catenin signalling pathway is one of the most important pathways in the initiation and progression of GIC^9^-^10^. Currently, the biomarker DKK-1 is being studied and is highly expressed in various GIC^11^-^12^. However, its diagnostic and prognostic efficacy for the most suitable subtype of GIC has not been determined. Even if there were previously published meta-analyses, the included original articles were not comprehensive enough, and no previous systematic review has provided a comprehensive overview with meta-regression and network meta-analysis for diagnostic and prognostic data.

## Methods

The network meta-analysis of this systematic review was structured according to the PRISMA (preferred reporting items for systematic reviews and meta-analyses) statement for diagnostic test accuracy and prognosis test significance^13^-^14^. This systematic review protocol was registered with the PROSPERO registry (No. CRD42020167910)^15^.

### Search Strategy and Selection Criteria

Four electronic databases (PubMed, Embase, the Cochrane Library and China National Knowledge Infrastructure) were searched using “Dickkopf-1”, “DKK-1”, “digestive”, “alimentary”, “gastrointestinal”, “cancer”, “carcinoma”, and “neoplasms” and their MeSH terms from inception to April 2020 (details are provided in **Table S1 in the Supplement**). Two researchers (L.H.Y. and H.F.H.) independently screened the titles and abstracts of original publications identified through the electronic search or reviewed the full text of potentially relevant articles as needed to retrieve the eligibility for inclusion in this systematic review and meta-analysis. Records published in the English or Chinese were included if they were described as retrospective and prospective observational studies. Any discrepancies were resolved by consensus with two experienced researchers (Z.Q.C. and Z.Y.S.). Studies were included if they met the following criteria: the original research type was diagnostic or prognostic. Patients were diagnosed with gastrointestinal carcinomas (including HCC, PC, GC, EPC, etc.), and provided data on the correlation between high and low DKK-1 expression and diagnosis or prognosis. Case reports, case series, studies without human data, and conference abstracts were excluded.

### Data Extraction and Quality Assessment

All data extraction from each study was independently undertaken by two researchers (J.X.W. and H.F.H.) using predesigned forms. For diagnostic-type research, basic characteristics (first author, publication year, country, cancer type, control type, case count, and control count), clinical features (biomarker test method, DKK-1 cut-off values, treatment, and all biomarkers used) and a 2×2 data table of true positive, false negative, true negative, and false positive results were used. For prognostic-type research, basic characteristics (first author, publication year, country, high/low expression count, cancer type, and control type), clinical features (patient treatment, research type, biomarker test method, DKK-1 cut-off values, and all biomarkers used) and histopathological features (tumour size, TNM stage, differentiation grade, lymphatic invasion, lymph node metastasis, vascular invasion, and distant metastasis) were assessed.

We used a modified Quality Assessment of Diagnostic Accuracy Studies 2 (QUADAS-2) tool to assess the quality of DKK-1 as one of the biomarker discovery studies^16^. QUADAS-2 consists of 4 domains, including patient selection, index test, reference standard, and flow of patients through the study, which could be included without high-risk options. We also used the Newcastle-Ottawa Scale (NOS) scale^17^ for observational study, which is used to determine the quality of DKK-1 as one of the prognostic indicators. NOS scores greater than 4 (max 10) can be included in this meta-analysis, and study quality was also independently assessed by two independent researchers.

### Outcomes and Analysis

For diagnostic-type research, high expression of DKK-1 alone or in combination with other biomarkers (DKK-1+AFP, DKK-1+CA19-9) was the indicator under investigation, and the sensitivity, specificity, positive likelihood ratio (PLR), negative likelihood ratio (NLR), diagnostic odds ratio (DOR) and the area under the receiver operating characteristic (ROC) curves were derived from diagnostic models of overall participants and subgroup studies for each type of carcinoma, including HCC, early HCC, PC, early PC, GC, EPC.

For prognostic-type research, high expression of DKK-1 alone) was the indicator under investigation. Histopathological stratification included tumour size (>5 cm vs ≤5 cm), TNM stage (III-IV vs I-II), differentiation grade (poor vs well/moderate), lymphatic invasion (yes/no), lymph node metastasis (yes/no), vascular invasion (yes/no) and distant metastasis (yes/no), which could also be subgrouped by cancer type (HCC, PC, GC, and EPC) and test method (IHC and ELISA).

### Synthesis of Evidence

To pool results from diagnostic-type research, we applied the hierarchical summary ROC model^18^ and obtained summary point estimates of the pairs of sensitivity and specificity, as well as DOR, PLR and NLR with their 95% confidence intervals (CIs). Summary estimates of the test accuracy were plotted in the ROC space together with the summary ROC curve^19^. To estimate the results from prognostic-type research, pooled odds ratios (ORs) or standardized mean differences (SMDs) with their 95% CIs were used to obtain a summary of the significant differences. The *I*^2^ statistic was used to assess the statistical heterogeneity among the included studies, when *I*^2^> 50% indicated high heterogeneity^20^, and regardless of heterogeneous results, random effects models were applied^21^. Subgroup analyses and meta-regression were performed on the basis of cancer type and test method. A *P* value less than 0.05 from the meta-regression indicated that this grouping method had a great impact on the overall results. If the network meta-analysis was demanded, the surface under the cumulative ranking (SUCRA) probabilities were used to rank them, and the higher SUCRA scores corresponded to a greater efficacy. Potential publication bias was evaluated by Deeks' asymmetry test for diagnostic-type outcomes and Harbord's test for prognostic-type outcomes^22^. In this study, MetaDisc (version 1.4) and STATAMP (version 14.0) software were used.

In addition, the quality of evidence for the diagnostic-type outcomes and prognostic-type research was assessed based on the GRADE system to estimate grading of recommendations, assessments, developments, and evaluations for diagnostic-type outcomes^23^, risk of bias (study limitations), imprecision, inconsistency, indirectness of study results, and publication bias for prognostic-type outcomes^24^.

## Results

### Study characteristics and Quality assessment

**Figure 1:**
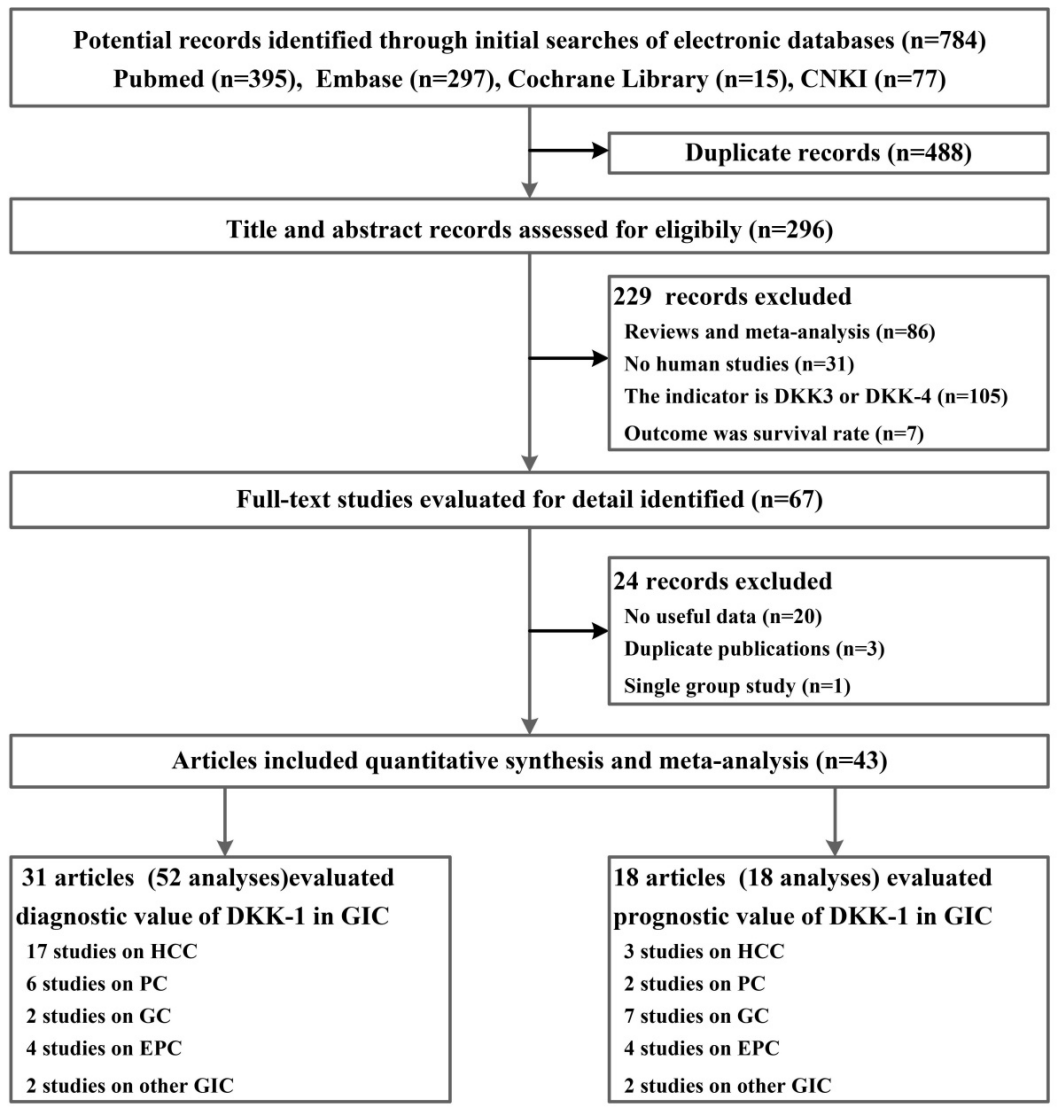
Flowchart summarizing publication search and study selection.

From 784 potential records identified through PubMed, Embase, the Cochrane Library, and China National Knowledge Infrastructure (CNKI), the literature systemic search yielded 43 publications^25^-^67^, including 9318 participants who met the eligibility criteria; of these participants, 31 of them had diagnostic data for DKK-1, and 18 records provided prognostic data for DKK-1(**Figure 1**). The number of subjects with or without GIC included in those publications ranged from 31 to 831. Among all included studies, HCC (n = 19), PC (n = 6), GC (n = 9), EPC (n = 6), CRC (n=1), intrahepatic cholangiocarcinoma (IHCC, n=1), and adenocarcinoma of the oesophagogastric junction (AEGJ, n=1) were researched. For the detection method of the DKK-1 protein, 31 publications utilized the enzyme-linked immunosorbent assay (ELISA) to detect the expression of DKK-1, and the other 12 articles used the immunohistochemical (IHC) method (**Table 1** and**Table S2** in the Supplement). Moreover, many of the studies we included not only used BKK-1 as a tumour biomarker but also explored the diagnostic synergistic effects of DKK-1 and other markers, including the combination of AFP in HCC and the combination of CA19-9 in PC. In addition, the results from baseline pairwise analysis showed that among the diagnostic tests, the GIC patient group showed a higher age and more males (**Table 1**). Quality assessments for the original diagnostic research by QUADAS-2 scales are summarized in **Table S3;** the original prognostic research by CASP scales are summarized in**Table S4**. All of our included publications had acceptable quality.

### Results of the combined diagnostic value of DKK-1

We first meta-analysed data for the diagnostic value of DKK-1 in overall GIC. The sensitivity and specificity were 0.70 (95% CI: 0.69-0.71) and 0.82 (0.81-0.83), respectively, with an AUC score of 0.8365, which means that the diagnostic value was moderate (**Table 2**). Therefore, we performed subgroup analysis and meta-regression to determine which type of GIC was most suitable for diagnosing DKK-1. No significant differences were found from the meta-regression (*P*=0.06) among different GIC. Of the eligible studies, 17 studies reported data on overall HCC, and the diagnostic value was also moderate, with the sub-subgroup study in the HCC group. When the control group was patients with LC±HBV±HCV from 7 studies, the diagnostic value was also moderate. When the intervention group was limited to patients with early HCC, the diagnostic value was also similar to that above. No significant results were found from the meta-regression in the above two sub-subgroups. For the diagnostic efficacy of DKK-1 in PC, the overall diagnostic value was higher, with an ROC score over 0.8818. For patients limited to early PC, the number of included studies was too small to determine the diagnostic value. For subgroup GIC types focusing on EPC and GC, low diagnostic value was detected (**Table 2**).

**Figure 2:**
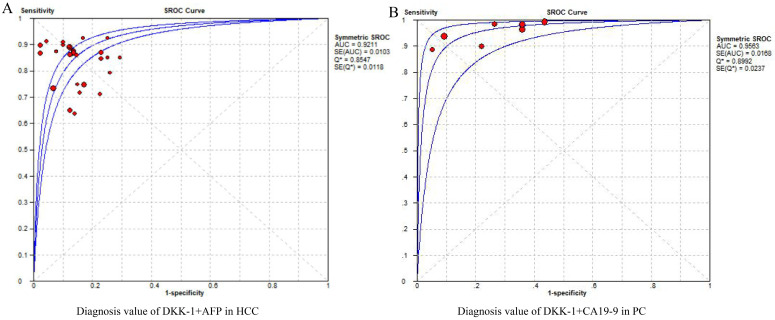
The pooled diagnostic accuracy of DKK-1+AFP in HCC diagnosis (A) and DKK-1+CA19-9 in PC diagnosis. DKK-1, dickkopf-1; HCC, hepatocellular carcinoma; PC, pancreatic carcinoma.

For the estimation of DKK-1's diagnostic efficacy, its combined diagnostic effect also needs to be considered because if the combined diagnostic efficiency was better, it also indicates that the expression of DKK-1 was more meaningful. For the combined diagnostic efficacy of DKK-1, we considered the diagnostic value of DKK-1+AFP in patients with HCC to be high, with an AUC of 0.9211. This is the combined diagnostic value of DKK-1 and AFP, which can also be said to improve the diagnostic efficiency of AFP from AUC=0.7941 (Table S5). Subgroups of the control group were patients with LC±HBV±HCV, and the diagnostic value was moderate. While the sub-subgroup of the intervention group was early HCC, the diagnostic value of DKK-1+AFP was higher than that of the overall HCC group, with an AUC score of 0.9109. For the combination of the diagnostic value of DKK-1+CA19-9, the diagnostic value was higher than that of DKK-1 alone (0.9563), which was the highest of all diagnostic values. For the diagnostic value of DKK-1 alone or in combination, no publication bias was found among overall, subgroup and sub-subgroup outcomes with a low to high GRADE (**Figure 2, Table 2**). Generally speaking, the diagnostic value of DKK-1+AFP was high in the HCC and early HCC groups. Additionally, DKK-1 alone or in combination with CA19-9 was effective in diagnosing PC.

### Results of combined prognostic value of DKK-1

Second, we performed subgroup meta-analysis and meta-regression for the histopathological stratification from subgroup analyses depending on the cancer type, and the test method used DKK-1 for the prognosis of GIC. For the outcome of tumour size, no significant differences were found in every subgroup; however, a low heterogeneity outcome was found in the GIC types of GC+EPC and GC alone. For the TNM meta-analysis, a significant difference was only found in the IHCC subgroup from only one study. This means that patients may have a more advanced TNM stage in the DKK-1 high expression group. For differentiation grade outcome, no significant differences could be found between high and low levels of DKK-1 expression. For the lymphatic invasion and lymph node metastasis groups, no significant results were found. When considering the vascular invasion results, no heterogeneity could be found in the overall LC group, the HCC group and the test method of the ELISA group, with no significant results, while no significant differences in distant metastasis outcome were found. No publication bias could be found in every group with low to moderate GRADE. In general, high DKK-1 expression may indicate a poor prognosis, especially in PC and IHCC. However, too few studies have been included in these two tumour subtypes, so we applied network meta-analysis to rank the analysis of tumour subtypes and test methods (**Table 3**).

**Figure 3:**
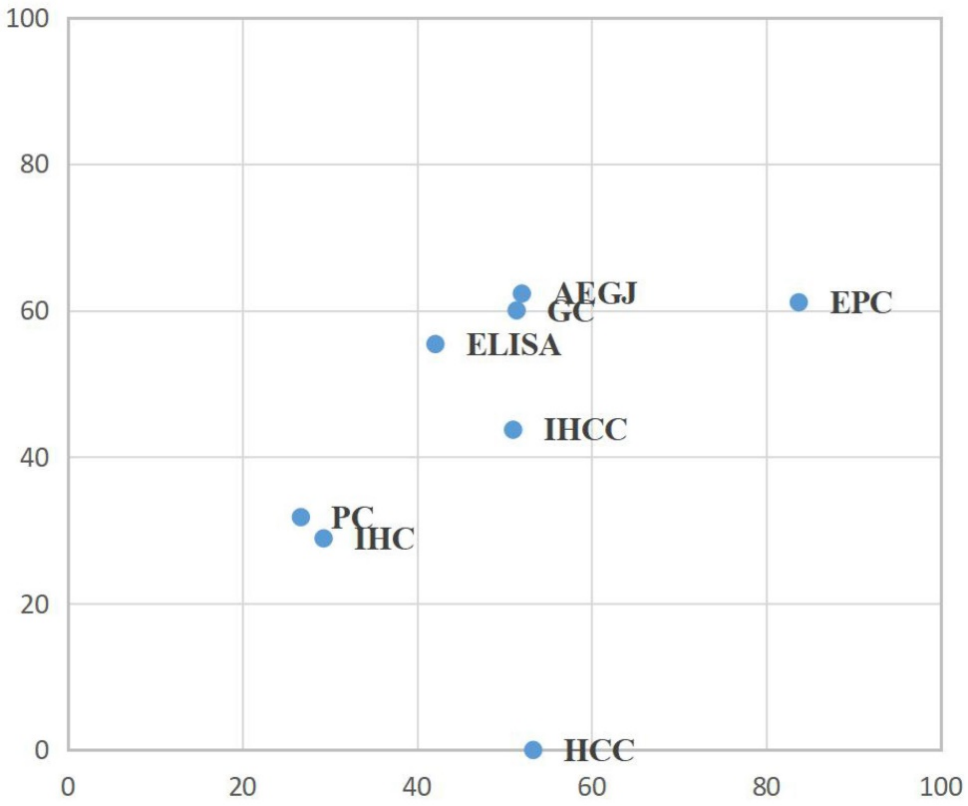
Surface under the cumulative ranking curve (SUCRA) rankings for TNM stage and lymph node metastasis in GIC patients. AEGJ, adenocarcinoma of oesophagogastric junction; CRC, colorectal carcinoma; DKK-1, dickkopf-1; ELISA, enzyme-linked immunosorbent assay; EPC, oesophageal carcinoma; GIC, gastrointestinal carcinomas; HCC, hepatocellular carcinoma; IHC, immunohistochemistry; IHCC, intrahepatic cholangiocarcinoma; LC, liver carcinomas; PC, pancreatic carcinomas.

To determine which was the most suitable subtype of GIC for the high expression of DKK-1 and which test method had a greater impact on the prognosis, we conducted a network meta-analysis for TNM stage and lymph node metastasis. For the combination of two outcomes, in EPC patients, high DKK-1 expression may be more related to histopathological stratification prognosis, but no significant difference exists (**Figure 3, Table S6**).

## Discussion

This manuscript focused on the diagnostic accuracy and prognostic significance of the biomarker protein DKK-1 in GIC. First, we conclude that DKK-1 has a moderate diagnostic value for overall GIC, a moderate diagnostic accuracy value for HCC and PC, and a low diagnostic accuracy value for EPC+GC. In addition, for the combined diagnostic value of DKK-1+AFP, a high diagnostic accuracy value could be determined in the HCC and early HCC groups. However, the diagnostic efficiency of DKK-1+CA19-9 was better than that of DKK-1 alone, with an AUC value above 0.95. Second, for the prognosis meta-analysis of histopathological stratification, we found that significant results occasionally appeared in the IHCC group. Furthermore, we found that EPC ranked best for the histopathological stratification of the prognosis, but there was no significant difference in these consequences. All of the above results show that DKK-1 has diagnostic accuracy and prognostic significance in GIC.

Generally, the accuracy of the diagnostic value was higher in HCC and PC, while the significance of prognostic efficacy was ranked first in EPC, which do not match. We think there are two reasons for this; first, the mismatch between diagnostic and prognostic data in the included publications. For example, 17 articles evaluated the DKK-1 diagnostic data of HCC, 5 articles evaluated the DKK-1 diagnostic data of PC, 3 articles evaluated the DKK-1 prognostic data of HCC, and 2 articles evaluated the DKK-1 prognostic data of PC. In addition, regarding the diagnostic efficacy of AFP in HCC and CA19-9 in PC, it can be said that DKK-1 improves these diagnostic capabilities (**Figure 2**). However, the combined efficacy was not analysed in prognostic data due to limited original research (**Table 3**). Therefore, for the combined prognosis data of HCC and PC, the included articles were too few to obtain clinical recommendation results.

Second, prognostic indicators of HCC and PC were relatively small, mainly due to the rapid development of HCC and PC, and few prognostic data can be provided. Moreover, there was a large difference between HCC and PC in the prognosis data from the included studies, so the data that can evaluate the prognosis indicators had clinical heterogeneity^68^; therefore, there may be no significant differences in the HCC group in our study. However, there are also data suggesting the role of DKK-1 overexpression in HCC development, which was consistent with the results provided by our predictive and diagnostic data. In addition, DKK-1 may have a role in the aggressiveness of pancreatic carcinoma cells, which could serve as a novel biomarker^69^. In addition, the progression of GC and EPC is slower, so the prognosis data could be merged in our meta-analysis. For the detection of DKK-1, the diagnostic test was used to detect the expression of DKK-1 in serum by ELISA. For the prognostic test, ELISA and IHC can both be utilized. Moreover, there were no significant differences between the effects of the two detection methods (**Table 3**, **Figure 3**).

Similar results could be found in previous incomplete publications for the diagnostic value of DKK-1^70^-^72^, and the combined diagnostic value of DKK-1+AFP could also be proven^70^-^71^. Moreover, Younis YS's research revealed that serum DKK-1 has higher sensitivity, specificity, and accuracy in early diagnosis of HCC than AFP^73^. The above evidence proved that there was a positive correlation among the invasion, aggressiveness and malignancy of tumours and the expression of DKK-1. The diagnosis and prognosis of DKK-1 are of great value. DKK-1 is an antagonist of the Wnt/β-catenin pathway^74^, and when investigating the correlation with cancer-related genes, high DKK-1 protein expression was associated with Wnt/β-catenin and its downstream signalling pathway. Therefore, DKK-1 could be used as a biomarker for predicting the progression of GIC^75^-^77^.

There are also some limitations among our systematic review and network meta-analysis. First, as previously mentioned, the results from the inclusion of diagnostic and prognostic data do not match, but we can still explain the reasons for such results. Second, the types of publications included are all observational studies, and the quality of the GRADE score results is not high, which may have a certain impact on the quality of our meta-analysis. Third, the results of significant differences were not found in our prognostic data. Our study included more studies of gastric cancer, but previous studies found significant differences^78^-^79^, proving that many clinical data are still needed for meta-analyses.

Our systematic review and meta-analyses found that DKK-1 alone provided modest diagnostic accuracy value for overall GIC, HCC and PC, whereas diagnostic accuracy were effective in EPC and GC. In addition, DKK-1+AFP provides high diagnostic accuracy value for HCC and early HCC. DKK-1+CA19-9 provides high diagnostic accuracy value for PC. Additionally, our results also suggest that DKK-1 expression is higher in front ranking EPCs in the network meta-analysis. All of the above results show that DKK-1 has good diagnostic accuracy, especially the combinations of DKK-1+AFP in HCC and DKK-1+CA19-9 in PC, whereas these had modest prognostic significance in GIC. Therefore, in clinical practice, we encourage patients suspected of HCC and PC to conduct DKK-1 detection to improve the diagnostic accuracy. Future head-to-head studies are warranted for DKK-1 expression in HCC and PC tissues to evaluate the prognostic value of DKK-1 in histopathological stratification to find the balance between diagnostic and prognostic data.

## Supplementary Material

Supplementary Table S1 Search strategies; Table S2 Main characteristics of DKK-1 in GIC;Table S3 Quality assessment of included studies by QUADAS-2 scales; Table S4 Quality assessment of included studies by Critical Appraisal Skills Programme (CASP) scales; Table S5 Summary estimates for the results from subgroup analyses depending on cancer type, cancer stage and control type using AFP alone or in combination with DKK-1 for the diagnosis of HCC; Table S6 SUCRA score from network meta-analysis for TNM stage and lymph node metastasis.

## Figures and Tables

**Table 1:** Main characteristics of the diagnostic and prognostic value of DKK-1 in GIC.

**Article type**	**Cancer type**	**Test method**	**No. of studies**	**No. of patients**
Diagnostic articles	HCC	ELISA	15	3962
		IHC	2	356
	PC	ELISA	3	574
		IHC	3	94
	EPC	ELISA	3	416
		IHC	1	288
	GC	ELISA	2	267
	AEGJ	ELISA	1	180
	CRC	ELISA	1	385
Prognostic articles	GC	IHC	7	1328
	EPC	ELISA	2	206
		IHC	2	220
	HCC	ELISA	2	172
		IHC	1	75
	IHCC	IHC	1	50
	PC	IHC	2	355
	AEGJ	ELISA	1	79
**Baseline Characteristics**		**Results**	**Heterogeneity**	**Significant**
Diagnostic	Sex	1.40 (1.17, 1.67)	0.071, 29.3%	Yes
	Age	0.47 (0.28, 0.66)	0.000, 85.6%	Yes
Prognostic	Sex	1.02 (0.76, 1.37)	0.286, 16.1%	No
	Age	-0.09 (-0.45, 0.27)	0.018, 70.3%	No

AEGJ, adenocarcinoma of oesophagogastric junction; CRC, colorectal carcinoma; DKK-1, dickkopf-1; ELISA, enzyme-linked immunosorbent assay; EPC, oesophageal carcinoma; GIC, gastrointestinal carcinomas; HCC, hepatocellular carcinoma; IHC, immunohistochemistry; IHCC, intrahepatic cholangiocarcinoma; LC, liver carcinomas; PC, pancreatic carcinomas.

**Table 2:** Summary estimates for the results from subgroup analyses depending on cancer type, cancer stage, control type used, and DKK-1 used alone or in combination for the diagnosis of GIC.

	No. of Studies (Analyses)	No. of participants (there is duplication)	Sensitivity (95% CI)	Specificity (95% CI)	Positive Likelihood Ratio (95% CI)	Negative Likelihood Ratio (95% CI)	Diagnostic Odds Ratio (95% CI)	Area Under the Curve	Mete-regression	Publication bias	GRADE
**DKK-1 used alone**											
**Overall**	31 (52)	11718	0.70 (0.69-0.71)	0.82 (0.81-0.83)	4.59 (3.50-6.01)	0.34 (0.29-0.39)	14.49 (9.72-21.60)	0.8365	0.06	0.893	High
**HCC**	17 (35)	9080	0.71 (0.69-0.72)	0.87 (0.86-0.88)	5.12 (4.08-6.41)	0.33 (0.29-0.37)	17.08 (12.83-22.74)	0.8515		0.208	High
**Compared with LC±HBV±HCV in HCC**	7 (16)	3964	0.71 (0.68-0.73)	0.86 (0.84-0.87)	4.79 (3.77-6.09)	0.34 (0.30-0.39)	14.98 (10.73-20.92)	0.8201	0.36	0.755	High
**Early HCC**	5 (11)	2885	0.73 (0.70-0.76)	0.90 (0.88-0.91)	6.17 (4.16-9.16)	0.31 (0.28-0.35)	22.14 (13.96-35.09)	0.8258	0.61	0.688	High
**PC**	6 (9)	1386	0.72 (0.69-0.75)	0.73 (0.70-0.76)	4.16 (2.15-8.04)	0.23 (0.12-0.45)	19.78 (5.35-73.08)	0.8818		0.712	Moderate
**Early PC**	1 (2)	154	0.85 (0.78-0.91)	0.77 (0.69-0.84)	3.69 (2.69-5.07)	0.19 (0.12-0.30)	19.23 (10.07-36.72)	-	0.28	-	Low
**GC+EPC**	6 (6)	1079	0.65 (0.61-0.69)	0.53 (0.49-0.57)	3.53 (1.15-10.88)	7.67 (3.93-14.95)	0.09 (0.04-0.19)	0.7358		0.289	Moderate
**DKK-1 in combination with other tumour marker**											
**DKK-1+AFP**											
**HCC**	12 (30)	7683	0.91 (0.82-0.96)	0.88 (0.87-0.89)	6.39 (5.26-7.77)	0.19 (0.16-0.23)	36.57 (26.30-50.86)	0.9211*		0.945	High
**Compared with LC±HBV±HCV in HCC**	5 (12)	2654	0.80 (0.78-0.82)	0.82 (0.79-0.84)	4.36 (3.69-5.17)	0.23 (0.17-0.29)	20.26 (14.22-28.87)	0.8876	0.09	0.582	High
**Early HCC**	5 (13)	3138	0.84 (0.82-086)	0.87 (0.85-0.88)	5.56 (4.20-7.37)	0.19 (0.14-0.26)	30.33 (18.37-50.09)	0.9109*	0.47	0.524	High
**DKK-1+CA19-9**											
**PC**	4 (7)	1109	0.96 (0.94-0.97)	0.72 (0.68-0.76)	3.70 (2.55-5.36)	0.06 (0.04-0.11)	80.46 (46.37-139.6)	0.9563*		0.881	High
**Early PC**	1 (2)	250	0.98 (0.94-1.00)	0.67 (0.58-0.75)	2.92 (2.26-3.76)	0.02 (0.01-0.09)	134.55 (31.18-580.67)	0.500	0.58	-	Moderate

AEGJ, adenocarcinoma of oesophagogastric junction; CRC, colorectal carcinoma; DKK-1, dickkopf-1; ELISA, enzyme-linked immunosorbent assay; EPC, oesophageal carcinoma; GIC, gastrointestinal carcinomas; HCC, hepatocellular carcinoma; IHC, immunohistochemistry; IHCC, intrahepatic cholangiocarcinoma; LC, liver carcinomas; PC, pancreatic carcinomas, *high diagnostic value.

**Table 3:** Summary estimates for the histopathological stratification from subgroup analyses depending on cancer subtype and DKK-1 test method used for the prognosis of GIC.

Histopathological stratification	Subgroup type	No. of studies	No. of participants	OR (95% CI)	*P, I^2^*	Meta-regression	Publication bias	GRADE
Tumour size (>5 cm vs ≤5 cm)	Overall	8	1070	1.12 (0.83, 1.52)	0.317, 14.4%		0.631	Moderate
	Cancer type					0.749		
	LC	4	295	1.22 (0.53, 2.83)	0.096, 52.8%		0.092	Low
	HCC	3	245	1.15 (0.35, 3.75)	0.043, 68.3%		0.204	Low
	IHCC	1	50	1.41 (0.44, 4.55)			-	Very low
	GC-EPC	4	775	1.06 (0.79, 1.42)	0.667, 0.0%^#^	-	0.319	Moderate
	GC	3	704	1.01 (0.74, 1.37)	0.822, 0.0%^#^		-	Moderate
TNM stage (III-IV vs I-II)	Overall	17	2366	1.80 (0.91, 3.59)	0.000, 91.0%		0.990	Moderate
	Cancer type					0.227		
	LC	3	218	2.24 (0.75, 6.70)	0.125, 52.0%		0.777	Very low
	HCC	2	168	1.54 (0.36, 6.54)	0.113, 60.3%		-	Very low
	IHCC	1	50	4.91 (1.22, 19.71)*	-		-	-
	GC-EPC	12	1793	1.56 (0.65, 3.75)	0.000, 93.3%		0.830	Moderate
	GC	7	1367	1.54 (0.40, 5.99)	0.000, 95.9%		0.727	Moderate
	EPC	4	426	1.64 (0.56, 4.77)	0.001, 81.4%		0.357	Moderate
	PC	2	355	3.16 (0.99, 10.07)	-		-	Very low
	Test method					0.609		
	ELISA	5	453	1.19 (0.49, 2.91)	0.006, 72.1%		0.556	Moderate
	IHC	12	1913	2.15 (0.89, 5.21)	0.000, 93.1%		0.990	Moderate
Differentiation grade (Poor vs Well/moderate)	Overall	11	1664	1.01 (0.70, 1.47)	0.018, 55.0%		0.900	Moderate
	Cancer type							
	LC(IHCC)	1	50	0.65 (0.18, 2.29)	-		-	Very low
	PC	2	355	0.49 (0.09, 2.72)	-		-	Very low
	GC-EPC	8	1259	1.17 (0.75, 1.82)	0.023, 59.1%		0.393	Moderate
	GC	4	959	1.07 (0.83, 1.81)	0.029, 66.7%		0.653	Moderate
	EPC	3	300	1.51 (0.55, 4.15)	0.099, 56.8%		0.912	Moderate
Lymphatic invasion (Yes/No)	Overall (GC)	3	464	0.54 (0.17, 1.74)	0.003, 89.13%		-	Moderate
Lymph node metastasis (Yes/No)	Overall	13	1898	1.10 (0.53, 2.29)	0.000, 90.2%	0.245	0.830	Moderate
	LC(IHCC)	1	50	5.18 (0.89, 30.09)	-		-	Very low
	PC	2	355	1.37 (0.21, 8.90)	-		-	Very low
	GC-EPC	10	1493	0.94 (0.38, 2.33)	0.000, 91.9%		0.756	Moderate
	GC	6	1239	0.91 (0.24, 3.52)	0.000, 95.0%		0.917	Moderate
	EPC	3	254	0.89 (0.23, 3.44)	0.004, 87.1%		0.730	Moderate
	Test method					0.789		
	ELISA	3	273	1.22 (0.33, 4.50)	0.005, 80.8%		0.410	Moderate
	IHC	10	1625	1.07 (0.44, 2.61)	0.000, 92.0%		0.974	Moderate
Vascular invasion (Yes/No)	Overall	10	1677	0.88 (0.31, 2.53)	0.000, 91.4%		0.840	Moderate
	Cancer type					0.143		
	LC	4	246	1.69 (0.83, 3.44)	0.419, 0.0%^#^		0.776	Moderate
	HCC	3	198	1.43 (0.68, 3.01)	0.686, 0.0%^#^		0.073	Moderate
	IHCC	1	48	8.29 (0.85, 3.01)	-		-	Very low
	GC	5	1120	0.53 (0.12, 2.42)	0.000, 95.3%		0.516	Moderate
	PC	1	311	0.65 (0.28, 1.50)	-			
	Test method					0.507		
	ELISA	2	121	1.67 (0.73, 3.83)	0.804, 0.0%^#^		-	Very low
	IHC	8	1556	0.72 (0.24, 2.19)	0.000, 92.2%		0.736	Moderate
Distant metastasis (Yes/No)	Overall	7	1427	0.79 (0.28, 2.24)	0.000, 85.2%		0.797	Moderate
	Cancer type					0.584		
	HCC	1	75	0.98 (0.28, 3.44)	-		-	Very low
	PC	1	311	0.45 (0.18, 1.14)	-			
	GC-EPC	5	1041	0.85 (0.20, 3.73)	0.000, 89.7%		0.808	Moderate
	GC	4	961	0.59 (0.11, 3.18)	0.000, 90.8%		0.617	Moderate

AEGJ, adenocarcinoma of oesophagogastric junction; CRC, colorectal carcinoma; DKK-1, dickkopf-1; ELISA, enzyme-linked immunosorbent assay; EPC, oesophageal carcinoma; GIC, gastrointestinal carcinomas; HCC, hepatocellular carcinoma; IHC, immunohistochemistry; IHCC, intrahepatic cholangiocarcinoma; LC, liver carcinomas; PC, pancreatic carcinomas, *Significant difference, ^#^No heterogeneity
