# Supplementary material for: Diagnosis Accuracy and Prognostic Significance of the Dickkopf-1 Protein in Gastrointestinal Carcinomas: Systematic Review and Network Meta-analysis
Source: J Cancer. 2020 Oct 18;11(24):7091–100. doi: 10.7150/jca.49970 (PMC7646173; doi:10.7150/jca.49970)
Supplement: Supplementary file 1 — Supplementary Table S1 Search strategies; Table S2 Main characteristics of DKK-1 in GIC;Table S3 Quality assessment of included studies by QUADAS-2 scales; Table S4 Quality assessment of included studies by Critical Appraisal Skills Programme (CASP) scales; Table S5 Summary estimates for the results from subgroup analyses depending on cancer type, cancer stage and control type using AFP alone or in combination with DKK-1 for the diagnosis of HCC; Table S6 SUCRA score from network meta-analysis for TNM stage and lymph node metastasis. [file jcav11p7091s1.pdf]

**Table S1 Search strategies**

## Search strategies for PubMed

#1. Dickkopf-1[All Fields] AND gastrointestinal[All Fields] AND ("carcinoma"[MeSH Terms] OR "carcinoma"[All Fields])

#2. DKK-1[All Fields] AND gastrointestinal[All Fields] AND ("carcinoma"[MeSH Terms] OR "carcinoma"[All Fields]) AND ("neoplasms"[MeSH Terms] OR "neoplasms"[All Fields] OR "cancer"[All Fields])

#3. #1 and #2

#4. DKK-1[All Fields] AND ("gastrointestinal neoplasms"[MeSH Terms] OR ("gastrointestinal"[All Fields] AND "neoplasms"[All Fields]) OR "gastrointestinal neoplasms"[All Fields] OR ("digestive"[All Fields] AND "cancer"[All Fields]) OR "digestive cancer"[All Fields])

#5. DKK-1[All Fields] AND alimentary[All Fields] AND ("neoplasms"[MeSH Terms] OR "neoplasms"[All Fields] OR "cancer"[All Fields])

#6. #4and #5

#7. #3 OR#6

## Search strategies for EMbase

#1 'Dickkopf-1$':ab,ti

#2 'DKK-1$':ab,ti

#3 'gastrointestinal':it

#4 'digestive':it

#5 'carcinoma':it

#6 ' cancer':it

#8 #1or#2

#9#3or#4

#10 #5or#6

#11 #5 and #6 and #7

## Search strategies for Cochrane library

#1. Dickkopf-1 or DKK-1, gastrointestinal cancer or digestive cancer

#2. MeSH descriptor

**Table S2 Main characteristics of DKK-1 in GIC**

**Table S2A Main characteristics of diagnosis value of DKK-1 in GIC**

| Study | Country | Cancer type | Control type | Case count(age, male/female) | Control count | Test method | DKK-1 Cut-off values | Treatment | All Mark used | Data extraction type | TP | FP | FN | TN |
|---|---|---|---|---|---|---|---|---|---|---|---|---|---|---|
| Zheng X, 2019[A1] | China | HCC | Benign liver diseases+Healthy control | 70（56.62±6.83） | 82 | ELISA | ≥2.0 ng/ml | Untreated | AFP+TK1+DKK1 | Reported in text | 47 | 7 | 23 | 75 |
| Zekri AR, 2020[A3] | Egypt | HCC | Cirrhotic +Cirrhotic +Healthy control | 78(58.27±9.7) | 160 | ELISA | 2000 pg/ml | Untreated | AFP+DKK1 | Estimated | 73 | 21 | 5 | 139 |
| Chen Z, 2019[1] | China | HCC | B1:Benign liver diseases B2:Healthy control | 120(49.6 ± 2.3,68/52) | B1:90(49.2 ± 2.0, 50/40) B2:80(48.6 ± 1.9,45/35) | ELISA | ≥2.0 ng/ml | Untreated | AFP+TK1+DKK1 | Reported in text | 66 | 54 | 15 | 75 |
| NAM Hassan(a), 2019[2] | Egypt | HCC | B1:liver cirrhosis B2:Healthy control | 40 | B1:24 B2:16 | ELISA | more than 331 pg/ml | Untreated | AFP+DKK1 | Estimated | 28 | 5 | 12 | 35 |
| NAM Hassan(b), 2019[2] | Egypt | HCC | liver cirrhosis | 40 | 24 | ELISA | more than 331 pg/ml | Untreated | AFP+DKK1 | Estimated | 28 | 3 | 12 | 21 |
| NAM Hassan(c), 2019[2] | Egypt | Early HCC | B1:liver cirrhosis B2:Healthy control | 20 | B1:24 B2:16 | ELISA | more than 331 pg/ml | Untreated | AFP+DKK1 | Estimated | 14 | 5 | 6 | 35 |

| | | | | | | | | | | | | | | |
|---|---|---|---|---|---|---|---|---|---|---|---|---|---|---|
| NAM Hassan(d), 2019[2] | Egypt | Early HCC | liver cirrhosis | 20 | 24 | ELISA | more than 331 pg/ml | Untreated | AFP+DKK1 | Estimated | 14 | 3 | 6 | 21 |
| Xi L(a), 2019[3] | China | HCC | B1:liver cirrhosis B2:HBV B3:Healthy control | 122(54.9 ± 6.0, 105/17) | B1:152(54.9 ±6.2,119/33) B2:105(55.5 ±6.7,81/24) B3:101(54.9 ±5.7,80/21) | ELISA | 0.431 | Untreated | AFP+CKAP4+DKK1 | Estimated | 80 | 8 | 42 | 350 |
| Xi L(b), 2019[3] | China | HCC | B1:liver cirrhosis B2:HBV B3:Healthy control | 76 | B1:152(54.9 ±6.2,119/33) B2:105(55.5 ±6.7,81/24) B3:101(54.9 ±5.7,80/21) | ELISA | 0.431 | Untreated | AFP+CKAP4+DKK1 | Estimated | 55 | 8 | 21 | 350 |
| Chen S, 2017[6] | China | PC | B1:Benign pancreatic disease B2:Healthy control | 122 | B1:58 B2:62 | ELISA | 2.0 ng/mL | Untreated | CA19-9+CA242+DKK1 | Estimated | 96 | 16 | 9 | 111 |
| Liu DJ,2017 [7] | China | PC | Healthy control | 311 | 311 | IHC | - | Unknown | DKK1 | Reported in text | 205 | 106 | 188 | 123 |
| Qin QF,2017 [8] | China | HCC | Benign liver diseases | 86 | 100 | ELISA | 2.0 ng/mL | Untreated | AFP+DCP+DKK1 | Reported in text | 63 | 23 | 56 | 44 |
| Xie H, 2017[9] | China | GC | B1:Benign gastrosia diseases B2:Healthy control | 75(60.3 ± 9.5,49/26) | B1:70(61.6 ± 6.9,48/22) B2:50(56.8 ± 7.7,39/11) | ELISA | 26.5 pg/mL | Untreated | CA72-4+TFF3+DKK1 | Estimated | 70 | 15 | 5 | 105 |

| | | | | | | | | | | | | | | |
|---|---|---|---|---|---|---|---|---|---|---|---|---|---|---|
| Xu Y,2017[10] | China | Adenocarcinoma of esophago-gastric junction | Healthy control | 79(38-782,65/14) | 101(32-75,78/23) | ELISA | 2615.9 pg/mL | Untreated | DKK1 | Estimated | 27 | 49 | 52 | 52 |
| Yao F H,2017[11] | China | HCC | B1:HBV group<br>B2:Healthy control | 117(55.9 ± 8.2, 81/36) | B1:80(52.0 ± 7.9, 50/30)<br>B2:80(53.8 ± 9.5 ,49/31) | ELISA | 2.153 μg/L | Untreated | DKK1+AFP+GP73 | Reported in text | 89 | 17 | 28 | 63 |
| Erdal H(a),2016[12] | Turkey | HCC | liver cirrhosis | 40(64.2 ± 8.9, 36/4) | 54(58.4 ± 10.0,33/21) | ELISA | 1.4μg/L | Untreated | AFP+DKK1+DKK3 | Estimated | 29 | 18 | 11 | 36 |
| Erdal H(b),2016[12] | Turkey | HCC | Healthy control | 40(64.2 ± 8.9, 36/4) | 39(58.4 ± 7.3, 10/29) | ELISA | 1.4μg/L | Untreated | AFP+DKK1+DKK3 | Estimated | 29 | 15 | 11 | 24 |
| Erdal H(c),2016[12] | Turkey | Early HCC | liver cirrhosis | 29 | 54(58.4 ± 10.0,33/21) | ELISA | 1.4μg/L | Untreated | AFP+DKK1+DKK3 | Estimated | 22 | 18 | 7 | 36 |
| Erdal H(d),2016[12] | Turkey | Early HCC | Healthy control | 29 | 39(58.4 ± 7.3, 10/29) | ELISA | 1.4μg/L | Untreated | AFP+DKK1+DKK3 | Estimated | 22 | 15 | 7 | 24 |
| Fouad YM,2016[13] | Egypt | HCC | B1:Chronic HCV group<br>B2:liver cirrhosis group | 50(59.3 ± 8.7, 34/16) | B1:20(42.7 ± 9.8, 13/7)<br>B2:20(54.3 ± 5.1,14/6) | ELISA | 1.53 ng/mL | Untreated | DKK1+AFP | Estimated | 34 | 4 | 16 | 36 |
| Mao L,2016[14] | China | HCC | B1:liver cirrhosis Group<br>B2:HBV group<br>B3:Healthy | 48(56.1, 37/11) | B1:20(52.3,16/4)<br>B2:20(37.8,13/7) | ELISA | 565ng/L | Untreated | DKK1 | Estimated | 45 | 18 | 3 | 42 |

| | | | | | | | | | | | | | | |
|---|---|---|---|---|---|---|---|---|---|---|---|---|---|---|
| | | | control | | B3:20(47.3,14/6) | | | | | | | | | |
| Su R,2016[15] | China | HCC | B1:HBV group<br>B2:liver cirrhosis Group<br>B3:Healthy control | 120(55.6 ± 14.2,84/36) | B1:60(47.7 ± 15.3, 32/28)<br>B2:60(53.5 ± 13.9, 36/24)<br>B3:60(50.5 ± 4.8, 35/25) | ELISA | 10.13ng/mL | Untreated | DKK1+AFP | Reported in text | 58 | 9 | 62 | 171 |
| Zhao P,2016[16] | China | HCC | B1:HBV group<br>B2:liver cirrhosis Group<br>B3:Healthy control | 60(55.6 ± 5.5, 54/6) | B1:40(44.2 ± 7.3, 31/9)<br>B2:40(58.6 ± 7.3, 27/13)<br>B3:40(48.6 ± 5.7, 28/12) | ELISA | 2.153μg/L | Untreated | DKK1+AFP | Estimated | 48 | 12 | 12 | 108 |
| Fu Y,2015[17] | China | HCC | adjacent tissue | 75(52.47 ± 10.62, 64/11) | 75 | IHC | More than 50% of the cells were positive | Surgery | C-MET+Golpll2+EZH2+DKKl | Reported in text | 63 | 24 | 12 | 51 |
| Ge T(a),2015[18] | USA | HCC | B1:liver cirrhosis Group<br>B2:HBV group<br>B3:Healthy control | 89 | B1:36<br>B2:65<br>B3:200 | ELISA | 1.31 ng/mL | Untreated | AFP+DKK1+OPN | Estimated | 71 | 32 | 18 | 269 |
| Ge T(b),2015[18] | USA | HCC | B1:liver cirrhosis Group<br>B2:HBV group | 89 | B1:36<br>B2:65 | ELISA | 1.31 ng/mL | Untreated | AFP+DKK1+OPN | Estimated | 71 | 7 | 18 | 94 |
| Ge T(c),2015[18] | USA | Early HCC | B1:liver cirrhosis Group<br>B2:HBV group | 56 | B1:36<br>B2:65<br>B3:200 | ELISA | 1.31 ng/mL | Untreated | AFP+DKK1+OPN | Estimated | 44 | 32 | 12 | 269 |

| | | | | | | | | | | | | | | |
|---|---|---|---|---|---|---|---|---|---|---|---|---|---|---|
| | | | B3:Healthy control | | | | | | | | | | | |
| Ge T(d),2015[18] | USA | Early HCC | B1:liver cirrhosis Group<br>B2:HBV group | 56 | B1:36<br>B2:65 | ELISA | 1.31 ng/mL | Untreated | AFP+DKK1+OPN | Estimated | 44 | 7 | 12 | 94 |
| Han SX(a),2015[19] | China | PC | B1:Benign pancreatic tumor<br>B2:Chronic pancreatitis<br>B3:Healthy control | 140(61.7 ± 10.8, 88/52) | B1:18(58.8 ± 12.1, 12/6)<br>B2:26(57.6 ± 11.3, 22/4)<br>B3:48(59.1 ± 11.0, 30/18) | ELISA | 39.3U/ml and 1560.02pg/ml | Untreated | DKK1+CA19-9 | Estimated | 125 | 19 | 15 | 73 |
| Han SX(b),2015[19] | China | PC | B1:Benign pancreatic tumor<br>B2:Chronic pancreatitis | 140(61.7 ± 10.8, 88/52) | B1:18(58.8 ± 12.1, 12/6)<br>B2:26(57.6 ± 11.3, 22/4) | ELISA | 39.3U/ml and 1560.02pg/ml | Untreated | DKK1+CA19-9 | Estimated | 125 | 12 | 15 | 32 |
| Han SX(c),2015[19] | China | Early PC | B1:Benign pancreatic tumor<br>B2:Chronic pancreatitis<br>B3:Healthy control | 62 | B1:18(58.8 ± 12.1, 12/6)<br>B2:26(57.6 ± 11.3, 22/4)<br>B3:48(59.1 ± 11.0, 30/18) | ELISA | 39.3U/ml and 1560.02pg/ml | Untreated | DKK1+CA19-9 | Estimated | 53 | 19 | 9 | 73 |
| Han SX(d),2015[19] | China | Early PC | B1:Benign pancreatic tumor<br>B2:Chronic pancreatitis | 62 | B1:18(58.8 ± 12.1, 12/6)<br>B2:26(57.6 ± 11.3, 22/4) | ELISA | 39.3U/ml and 1560.02pg/ml | Untreated | DKK1+CA19-9 | Estimated | 53 | 12 | 9 | 32 |
| Peng Y,2015[22] | China | EPC | Healthy control | 126(40-80, 102/24) | 60(37-76,47/13) | ELISA | | Non-surgery | DKK1+P53 | Estimated | 78 | 3 | 48 | 57 |
| Wang | China | PC | Benign | 44 | 19 | IHC | More | Untre | DKK1+ | Repor | 30 | 1 | 14 | 18 |

| | | | | | | | | | | | | | | |
|---|---|---|---|---|---|---|---|---|---|---|---|---|---|---|
| Q,2014[ 25] | | | pancreatic tumor | | | | than 50% of the cells were positive | ated | CA19-9 | ted in text | | | | |
| Zhou Y,2014[ 26] | China | PC | Healthy control | 50(65.0 4 ± 13.55, 31/19) | 50(53.42 ± 10.66, 27/23) | ELIS A | 22.5μ g / L | Untre ated | DKK1+ CA19-9 | Estim ated | 26 | 4 | 24 | 46 |
| Peng L,2013[ 27] | China | HCC | B1:HBV group B2:liver cirrhosis Group | 35 | B1:38 B2:105 | ELIS A | 2.13 ng/mL | Untre ated | AFP+D KK1 | Estim ated | 28 | 29 | 7 | 117 |
| Yang H(a),201 3[28] | China | Early stage HCC | liver cirrhosis Group | 104(54. 5,91/13 ) | 102(45,77/25) | IHC | 6.25 ng/mL | Untre ated | AFP+D KK1 | Estim ated | 61 | 21 | 43 | 81 |
| Yang H(b),201 3[28] | China | Early stage HCC | liver cirrhosis Group | 80(51.5, 71/9) | 72(43,58/14) | IHC | 6.25 ng/mL | Surger y | AFP+D KK1 | Estim ated | 44 | 13 | 36 | 59 |
| Shen Q(a),201 2[31] | China | HCC | B1:HBV group B2:liver cirrhosis Group B3:Healthy control | 424 | B1:98 B2:96 B3:213 | ELIS A | 0.03 ng/mL | Untre ated | AFP+D KK1 | Estim ated | 293 | 38 | 131 | 369 |
| Shen Q(b),201 2[31] | China | HCC | B1:HBV group B2:liver cirrhosis Group | 424 | B1:98 B2:96 | ELIS A | 0.03 ng/mL | Untre ated | AFP+D KK1 | Estim ated | 293 | 30 | 131 | 164 |
| Shen Q(c),201 2[31] | China | HCC | B1:HBV group B2:liver cirrhosis Group B3:Healthy control | 209 | B1:73 B2:72 B3:99 | ELIS A | 0.03 ng/mL | Untre ated | AFP+D KK1 | Estim ated | 149 | 31 | 60 | 213 |

| | | | | | | | | | | | | | | |
|---|---|---|---|---|---|---|---|---|---|---|---|---|---|---|
| Shen Q(d),2012[31] | China | HCC | B1:HBV group B2:liver cirrhosis Group | 209 | B1:73 B2:72 | ELISA | 0.03 ng/mL | Untreated | AFP+DKK1 | Estimated | 149 | 14 | 60 | 131 |
| Zhang Y,2012[33] | China | A1:EPC A2:precancerous lesions | Healthy control | A1:72 A2:13 | 30 | ELISA | 13.4μg/L | Untreated | DKK1 | Reported in text | 63 | 1 | 22 | 29 |
| Soydinc HO,2011[34] | Turkey | CRC | Healthy control | 295(59) | 90(50) | ELISA | 29.36 ng/mL | Untreated | DKK1 | Estimated | 175 | 45 | 120 | 45 |
| Zhou Y, 2011[36] | China | PC | Benign pancreatic disease | 24 | 7 | IHC | More than 5% of the cells were positive | Untreated | DKK1 | Reported in text | 21 | 0 | 3 | 7 |
| Zhang X,2010[37] | China | GC | Healthy control | 34(28-87, 23/11) | 38(22-75, 25/13) | ELISA | 3.539μg/L | Untreated | DKK1 | Estimated | 21 | 6 | 13 | 32 |
| Li B,2009[38] | China | EPC | Healthy control | 80(45-79,68/12) | 35 | ELISA | 14.54ng/mL | Untreated | DKK1 | Reported in text | 53 | 6 | 27 | 29 |
| Yamabuki T,2007[40] | Japan | EPC | Healthy control | 81 | 207 | IHC | 3.3μg/L | Untreated | DKK1 | Reported in text | 51 | 197 | 30 | 10 |

A:Cancer case group; B: Control case group; ELISA:enzyme-linked immunosorbent assay;IHC: Immunohistochemistry;

HCC:hepatocellular carcinoma; PC:pancreatic carcinoma ; GC:gastric carcinoma ; EPC:esophageal carcinoma; CRC: Colorectal carcinoma

FN = fasle negative; FP = false positive; TN = true negative; TP = true positive.

**29researches, 46analysis**

**Table S2B Main characteristics of prognostic value of DKK-1 in GIC**

| Study | Country | High/Low(age,male/female) | Cancer type | Control type | Patients treatment | Research type | Test method | Cut-off values | All Mark used | Tumor size(<5/>5cm) | TNM stage(I+II/III+IV) | Differentiation grade (Well and moderate/Poor ) | Lymphatic invasion (Yes/No) | Lymph node metastasis(Yes/No) | Vascular invasion(Yes/No) | Distant metastasis(Yes/No) | Data extraction type |
|---|---|---|---|---|---|---|---|---|---|---|---|---|---|---|---|---|---|
| Hu W,2020[A2] | China | H:21 L:26 | GC | adjacent tissue+Healthy control | Surgery | Case-control study | IHC | Unknown | DKK-1 | H:14/7 L:18/8 | H:3/18 L:17/9 | | | H:18/3 L:15/12 | | | |
| Gu L,2018 [4] | China | H:28(14/14) L:132(80/52) | GC | B1: adjacent tissue B2:Normal gastric mucosa | | Case-control study | IHC | More than 25% of the cells were positive | OPN, sLAG-3, DKK-1 | | H:18/10 L:10/82 | H:10/18 L:80/52 | - | H:16/12 L:84/48 | - | - | Reported in text |
| Hong SA, 2018[5] | Korea | H:73(53/20 L:85(60/25) | GC | - | Surgery | Case-control study | IHC | More than 60 H scores, | DKK1 expression with ß-catenin positivity | | H:27/46 L:41/44 | H:26/47 L:20/65 | H:62/11 L:65/20 | - | H:7/66 L:5/80 | | Reported in text |
| Liu DJ, 2017[7] | China | H:205(122/83) L:106(54/52) | PC | Healthy control | Unknown | Case-control study | IHC | Unknown | DKK-1 | | H:20/185 L:19/87 | H:128/77 L:66/40 | | H:120/85 L:75/31 | H:182/23 L:98/8 | H:181/24 L:100/6 | Reported in text |
| Xu | China | H:27(22 | Adenoc | - | Surger | Case-c | ELIS | 2615.9 | DKK-1 | H:10/1 | H:5/22 | - | - | H:22/5 | - | H:8/19 | Estima |

| | | | | | | | | | | | | | | | | | |
|---|---|---|---|---|---|---|---|---|---|---|---|---|---|---|---|---|---|
| Y,2017 [10] | | /5) L:52(43/9) | arcinoma of esophago-gastric junction | | y | ontrol study | A | pg/mL | | 5 L:25/21 | L:9/43 | | | L:32/10 | | L:5/47 | ted |
| Fu Y,2015 [17] | China | H:63(51.2±11.6, 56/7) L:12(52.7 ± 10.5,8/4) | HCC | adjacent tissue | Surgery | Case-control study | IHC | More than 50% of the cells were positive | C-MET+Golpll2+EZH2+DKKl | H:42/21 L:6/6 | | | | | H：7/56 L：2/10 | H:26/37 L:5/7 | Reported in text |
| Ma G,2015[20] | China | H:133(102/31) L:195(133/62) | GC | - | Surgery+chemotherapy | Case-control study | IHC | More than 50% of the cells were positive | DKKl | H:76/57 L:116/79 | H:75/36 L:71/146 | H:35/92 L:61/137 | | H:52/81 L:150/45 | H:27/106 L:100/95 | H:6/127 L:40/155 | Reported in text |
| Ma X,2015[21] | China | H:18(13/5) L:54(43/11) | HCC | - | Surgery | Case-control study | ELISA | 1769 pg/mL | DKKl | H:12/6 L:33/21 | H:15/3 L:42/12 | | | | H:10/8 L:22/32 | | Reported in text |
| Peng Y,2015 [22] | China | H:57(42/15) L:69(60/9) | EPC | - | Non-surgery | Case-control study | ELISA | More than 50% of the cells were positive | DKK1+P53 | | H:35/22 L:28/41 | | | H:33/24 L:51/16 | | | Reported in text |
| Tan J,2015 | China | H:35(28/7) | EPC | - | Surgery | Case-control | IHC | More than 50% | DKK1 | | H:19/16 | H:24/11 | | H:17/18 | | | Reported in |

| | | | | | | | | | | | | | | | | | |
|---|---|---|---|---|---|---|---|---|---|---|---|---|---|---|---|---|---|
| [23] | | L:15(10/5) | | | | study | | of the cells were positive | | | L:14/1 | L:14/1 | | L:10/5 | | | text |
| Cai L,2014 [24] | China | H:27(60 ± 14.93, 19/8) L:126(58.11 ± 12.07, 83/43) | GC | - | Surgery | Case-control study | IHC | 60 pg/mL | DKK1+CEA+CA19-9 | | H:15/12 L:100/26 | | H:11/16 L:88/38 | H:22/5 L:117/9 | H:16/11 L:114/12 | H:22/5 L:117/9 | Reported in text |
| Wang Q,2014[25] | China | H:30 L:14 | PC | - | Untreated | Case-control study | IHC | More than 50% of the cells were positive | DKK1+CEA+CA19-9 | | H:8/22 L:10/4 | H:15/15 L:2/12 | | H:24/6 L:7/7 | | | Reported in text |
| Gao C,2012[29] | China | H:195(57.2 ± 9.6, 133/62) L:133(61.9 ± 11.8,102//31) | GC | - | Surgery | Case-control study | IHC | More than 50% of the cells were positive | DKK1 | H:116/79 L:76/57 | H:49/146 L:97/36 | H:61/132 L:35/88 | | H:150/45 L:52/81 | H:100/95 L:27/106 | H:40/155 L:6/127 | Reported in text |
| Lee HS,2012[30] | Korea | H:27(60 ± 14.93,19/8) L:126(58.11 ± | GC | Healthy control | Surgery | Case-control study | IHC | 60 pg/mL | DKK1+CEA+CA19-9 | | H:15/12 L:100/26 | | H:11/16 L:88/38 | H:13/14 L:81/45 | H:16/11 L:114/12 | H:22/5 L:117/9 | Reported in text |

| | | | | | | | | | | | | | | | | | |
|---|---|---|---|---|---|---|---|---|---|---|---|---|---|---|---|---|---|
| | | 12.07, 83/43) | | | | | | | | | | | | | | | |
| Zhang X,2012[32] | China | H:19(10/9) L:31(18/13) | Intrahepatic cholangiocarcinoma | Healthy control | Untreated | Case-control study | IHC | More than 25% of the cells were positive | DKK1 | H:7/12 L:14/17 | H:11/8 L:27/4 | H:14/5 L:20/11 | | H:5/14 L:2/29 | H:4/14 L:1/29 | | Reported in text |
| Tung EK, 2011[35] | China | H:23(19/4) L:77(63/14) | HCC | - | Untreated | Case-control study | ELISA | 1500 pg/mL | DKK1 | H:5/17 L:39/37 | H:4/18 L:30/44 | | | | H:6/6 L:15/22 | | Reported in text |
| Li B,2009[38] | China | H:53 L:27 | EPC | Healthy control | Untreated | Case-control study | ELISA | 14.54ng/mL | DKK1+CEA+CYFRA21-1 | | H:17/36 L:16/11 | H:40/13 L:18/9 | | H:43/10 L:15/12 | | | Reported in text |
| Makino T,2009[39] | Japan | H:72(63/9) L:98(89/9) | EPC | - | Chemotherapy | Case-control study | IHC | More than 10% of the cells were positive | DKK1 | | H:28/44 L:45/53 | H:13/59 L:28/70 | | | | | Reported in text |

H:High expresion group; L: Low expresion group; ELISA:enzyme-linked immunosorbent assay;IHC: Immunohistochemistry;
HCC:hepatocellular carcinoma; PC:pancreatic carcinoma ; GC:gastric carcinoma ; EPC:esophageal carcinoma; CRC: Colorectal carcinoma

**Table S3 Quality assessment of included of studies by QUADAS-2 scales.**

| Study | PATIENT SELECTION | | | | INDEX TEST(S) | | | REFERENCE STANDARD | | | FLOW AND TIMING | | | | |
|---|---|---|---|---|---|---|---|---|---|---|---|---|---|---|---|
| | Was a consecutive or random sample of patients enrolled? | Was a case-control design avoided? | Did the study avoid inappropriate exclusions? | **Is there concern that the included patients do not match the review question?** | Were the index test results interpreted without knowledge of the results of the reference standard? | If a threshold was used, was it pre-specified? | **Is there concern that the index test, its conduct, or interpretation differ from the review question?** | Is the reference standard likely to correctly classify the target condition? | Were the reference standard results interpreted without knowledge of the results of the index test? | **Is there concern that the target condition as defined by the reference standard does not match the review question?** | Was there an appropriate interval between index test(s) and reference standard? | Did all patients receive a reference standard? | Did patients receive the same reference standard? | Were all patients included in the analysis? | **Could the patient flow have introduced bias?** |
| Chen Z, 2019[1] | Yes | Yes | Unclear | Unclear | Yes | Unclear | Unclear | Yes | Unclear | Unclear | Unclear | Yes | Yes | Yes | Unclear |
| NAM Hassan, 2019[2] | Yes | Yes | Unclear | Unclear | Yes | Unclear | Unclear | Yes | Unclear | Unclear | Unclear | Yes | Yes | Yes | Unclear |
| Xi L, 2019[3] | Yes | Yes | Unclear | Unclear | Yes | Unclear | Unclear | Yes | Unclear | Unclear | Unclear | Yes | Yes | Yes | Unclear |
| Chen S, 2017[6] | Yes | Yes | Unclear | Unclear | Yes | Unclear | Unclear | Yes | Unclear | Unclear | Unclear | Yes | Yes | Yes | Unclear |
| Qin QF,2017[8] | Yes | Yes | Unclear | Unclear | Yes | Unclear | Unclear | Yes | Unclear | Unclear | Unclear | Yes | Yes | Yes | Unclear |
| Xie H, 2017[9] | Yes | Yes | Unclear | Unclear | Yes | Unclear | Unclear | Yes | Unclear | Unclear | Unclear | Yes | Yes | Yes | Unclear |
| Xu Y,2017[10] | Yes | Yes | Unclear | Unclear | Yes | Unclear | Unclear | Yes | Unclear | Unclear | Unclear | Yes | Yes | Yes | Unclear |
| Yao F H,2017[11] | Yes | Yes | Unclear | Unclear | Yes | Unclear | Unclear | Yes | Unclear | Unclear | Unclear | Yes | Yes | Yes | Unclear |
| Erdal H,2016[12] | Yes | Yes | Unclear | Unclear | Yes | Unclear | Unclear | Yes | Unclear | Unclear | Unclear | Yes | Yes | Yes | Unclear |
| Fouad YM,2016[13] | Yes | Yes | Unclear | Unclear | Yes | Unclear | Unclear | Yes | Unclear | Unclear | Unclear | Yes | Yes | Yes | Unclear |

| | | | | | | | | | | | | | | | |
|---|---|---|---|---|---|---|---|---|---|---|---|---|---|---|---|
| Mao L,2016[14] | Yes | Yes | Unclear | Unclear | Yes | Unclear | Unclear | Yes | Unclear | Unclear | Unclear | Yes | Yes | Yes | Unclear |
| Su R,2016[15] | Yes | Yes | Unclear | Unclear | Yes | Unclear | Unclear | Yes | Unclear | Unclear | Unclear | Yes | Yes | Yes | Unclear |
| Zhao P,2016[16] | Yes | Yes | Unclear | Unclear | Yes | Unclear | Unclear | Yes | Unclear | Unclear | Unclear | Yes | Yes | Yes | Unclear |
| Fu Y,2015[17] | Yes | Yes | Unclear | Unclear | Yes | Unclear | Unclear | Yes | Unclear | Unclear | Unclear | Yes | Yes | Yes | Unclear |
| Ge T,2015[18] | Yes | Yes | Unclear | Unclear | Yes | Unclear | Unclear | Yes | Unclear | Unclear | Unclear | Yes | Yes | Yes | Unclear |
| Han SX,2015[19] | Yes | Yes | Unclear | Unclear | Yes | Unclear | Unclear | Yes | Unclear | Unclear | Unclear | Yes | Yes | Yes | Unclear |
| Peng Y,2015[22] | Yes | Yes | Unclear | Unclear | Yes | Unclear | Unclear | Yes | Unclear | Unclear | Unclear | Yes | Yes | Yes | Unclear |
| Wang Q,2014[25] | Yes | Yes | Unclear | Unclear | Yes | Unclear | Unclear | Yes | Unclear | Unclear | Unclear | Yes | Yes | Yes | Unclear |
| Zhou Y,2014[26] | Yes | Yes | Unclear | Unclear | Yes | Unclear | Unclear | Yes | Unclear | Unclear | Unclear | Yes | Yes | Yes | Unclear |
| Peng L,2013[27] | Yes | Yes | Unclear | Unclear | Yes | Unclear | Unclear | Yes | Unclear | Unclear | Unclear | Yes | Yes | Yes | Unclear |
| Yang H,2013[28] | Yes | Yes | Unclear | Unclear | Yes | Unclear | Unclear | Yes | Unclear | Unclear | Unclear | Yes | Yes | Yes | Unclear |
| Shen Q,2012[31] | Yes | Yes | Unclear | Unclear | Yes | Unclear | Unclear | Yes | Unclear | Unclear | Unclear | Yes | Yes | Yes | Unclear |
| Zhang Y,2012[33] | Yes | Yes | Unclear | Unclear | Yes | Unclear | Unclear | Yes | Unclear | Unclear | Unclear | Yes | Yes | Yes | Unclear |
| Soydinc HO,2011[34] | Yes | Yes | Unclear | Unclear | Yes | Unclear | Unclear | Yes | Unclear | Unclear | Unclear | Yes | Yes | Yes | Unclear |
| Zhou Y, 2011[36] | Yes | Yes | Unclear | Unclear | Yes | Unclear | Unclear | Yes | Unclear | Unclear | Unclear | Yes | Yes | Yes | Unclear |
| Zhang X,2010[37] | Yes | Yes | Unclear | Unclear | Yes | Unclear | Unclear | Yes | Unclear | Unclear | Unclear | Yes | Yes | Yes | Unclear |
| Li B,2009[38] | Yes | Yes | Unclear | Unclear | Yes | Unclear | Unclear | Yes | Unclear | Unclear | Unclear | Yes | Yes | Yes | Unclear |
| Yamabuki T,2007[40] | Yes | Yes | Unclear | Unclear | Yes | Unclear | Unclear | Yes | Unclear | Unclear | Unclear | Yes | Yes | Yes | Unclear |
| Zheng X, 2019[A1] | Yes | Yes | Unclear | Unclear | Yes | Unclear | Unclear | Yes | Unclear | Unclear | Unclear | Yes | Yes | Yes | Unclear |
| Zekri AR, 2020[A3] | Yes | Yes | Unclear | Unclear | Yes | Unclear | Unclear | Yes | Unclear | Unclear | Unclear | Yes | Yes | Yes | Unclear |

**Table S4 Quality assessment of included of studies by Critical Appraisal Skills Programme (CASP) scales.**

| Study, Year | 1.Did the study address a clearly focused issue? | 2.Was the cohort recruited in an acceptable way? | 3.Was the exposure accurately measured to minimise bias? | 4.Was the outcome accurately measured to minimise bias? | 5. (a) Have the authors identified all important confounding factors? | 5. (b) Have they taken account of the confounding factors in the design and/or analysis? | 6. (a) Was the follow up of subjects complete enough? | 6. (b) Was the follow up of subjects long enough? | 7. What are the results of this study? | 8. How precise are the results? | 9. Do you believe the results? | 10.Can the results be applied to the local population? | 11. Do the results of this study fit with other available evidence? | 12. What are the implications of this study for practice? |
|---|---|---|---|---|---|---|---|---|---|---|---|---|---|---|
| Gu L,2018[4] | Yes | Yes | Yes | Yes | Can’t tell | Can’t tell | Can’t tell | Can’t tell | Yes | Can’t tell | Yes | Yes | Yes | Yes |
| Hong SA, 2018[5] | Yes | Yes | Yes | Yes | Can’t tell | Can’t tell | Yes | Yes | Yes | Can’t tell | Yes | Yes | Yes | Yes |
| Xu Y,2017[10] | Yes | Yes | Yes | Yes | Can’t tell | Can’t tell | Can’t tell | Can’t tell | Yes | Can’t tell | Yes | Yes | Yes | Yes |
| Fu Y,2015[17] | Yes | Yes | Yes | Yes | Can’t tell | Can’t tell | Can’t tell | Can’t tell | Yes | Can’t tell | Yes | Yes | Yes | Yes |
| Ma G,2015[20] | Yes | Yes | Yes | Yes | Can’t tell | Can’t tell | Yes | Can’t tell | Yes | Can’t tell | Yes | Yes | Yes | Yes |
| Ma X,2015[21] | Yes | Yes | Yes | Yes | Can’t tell | Can’t tell | Yes | Can’t tell | Yes | Can’t tell | Yes | Yes | Yes | Yes |
| Peng Y,2015[22] | Yes | Yes | Yes | Yes | Can’t tell | Can’t tell | Can’t tell | Can’t tell | Yes | Can’t tell | Yes | Yes | Yes | Yes |
| Tan J,2015[23] | Yes | Yes | Yes | Yes | Can’t tell | Can’t tell | Can’t tell | Can’t tell | Yes | Can’t tell | Yes | Yes | Yes | Yes |
| Cai L,2014[24] | Yes | Yes | Yes | Yes | Can’t tell | Can’t tell | Can’t tell | Can’t tell | Yes | Can’t tell | Yes | Yes | Yes | Yes |

| | | | | | | | | | | | | | | |
|---|---|---|---|---|---|---|---|---|---|---|---|---|---|---|
| Wang Q,2014[25] | Yes | Yes | Yes | Yes | Can’t tell | Can’t tell | Yes | No | Yes | Can’t tell | Yes | Yes | Yes | Yes |
| Gao C,2012[29] | Yes | Yes | Yes | Yes | Can’t tell | Can’t tell | Yes | Can’t tell | Yes | Can’t tell | Yes | Yes | Yes | Yes |
| Lee HS,2012[30] | Yes | Yes | Yes | Yes | Can’t tell | Can’t tell | Yes | Yes | Yes | Can’t tell | Yes | Yes | Yes | Yes |
| Zhang X,2012[32] | Yes | Yes | Yes | Yes | Can’t tell | Can’t tell | Can’t tell | Yes | Yes | Can’t tell | Yes | Yes | Yes | Yes |
| Tung EK, 2011[35] | Yes | Yes | Yes | Yes | Can’t tell | Can’t tell | Can’t tell | Can’t tell | Yes | Can’t tell | Yes | Yes | Yes | Yes |
| Li B,2009[38] | Yes | Yes | Yes | Yes | Can’t tell | Can’t tell | Yes | Yes | Yes | Can’t tell | Yes | Yes | Yes | Yes |
| Makino T,2009[39] | Yes | Yes | Yes | Yes | Can’t tell | Can’t tell | Yes | Yes | Yes | Can’t tell | Yes | Yes | Yes | Yes |
| Hu W,2020[A2] | Yes | Yes | Yes | Yes | Can’t tell | Can’t tell | Yes | Yes | Yes | Can’t tell | Yes | Yes | Yes | Yes |

| =- | No.of Analyses | No. of participants | Sensitivity, (95% CI) | Specificity, (95% CI) | Positive Likelihood Ratio (95% CI) | Negative Likelihood Ratio (95% CI) | Diagnostic Odds Ratio (95% CI) | Area Under the Curve | Mete-regression | Publication bias | GRADE |
|---|---|---|---|---|---|---|---|---|---|---|---|
| | | | | | **DKK-1+AFP** | | | | | | |
| **HCC** | 12 (30) | 7683 | 0.91 (0.82-0.96) | 0.88 (0.87-0.89) | 6.39 (5.26-7.77) | 0.19 (0.16-0.23) | 36.57 (26.30-50.86) | 0.9211* | | 0.945 | High |
| **Compared with LC±HBV±HCV in HCC** | 5(12) | 2654 | 0.80(0.78-0.82) | 0.82(0.79-0.84) | 4.36(3.69-5.17) | 0.23(0.17-0.29) | 20.26(14.22-28.87) | 0.8876 | 0.14 | 0.582 | High |
| **Early HCC** | 5(13) | 3138 | 0.84(0.82-086) | 0.87(0.85-0.88) | 5.56(4.20-7.37) | 0.19(0.14-0.26) | 30.33(18.37-50.09) | 0.9109* | 0.88 | 0.524 | High |
| | | | | | | **AFP** | | | | | |
| **HCC** | 11(17) | 4872 | 0.65 (0.63-0.67) | 0.73 (0.71-0.75) | 3.17 (2.35-4.28) | 0.41(0.35-0.49) | 8.30(5.33-12.93) | 0.7941 | | 0.180 | High |
| | | | | | | **DKK-1** | | | | | |
| **HCC** | 17 (35) | 9080 | 0.71 (0.69-0.72) | 0.87 (0.86-0.88) | 5.12 (4.08-6.41) | 0.33 (0.29-0.37) | 17.08 (12.83-22.74) | 0.8515 | | 0.208 | High |

**Table S6 SUCRA score from network meta-analysis for TNM stage and lymph node metastasis.**

| Sub-type | TNM stage | Lymph node metastasis |
|---|---|---|
| EPC | 83.7 | 61.1 |
| GC | 51.4 | 60 |
| AEGJ | 52 | 62.3 |
| HCC | 53.3 | 0 |
| IHCC | 51 | 43.7 |
| PC | 26.7 | 31.8 |
| IHC | 83.7 | 61.1 |
| ELISA | 51.4 | 60 |
| Low group | 38.2 | 9 |
